# Effect of interval between preoperative radiotherapy and surgery on clinical outcome and radiation proctitis in rectal cancer from FOWARC trial

**DOI:** 10.1002/cam4.2755

**Published:** 2019-12-12

**Authors:** Yi‐Kan Cheng, Qi‐Yuan Qin, Xiao‐Yan Huang, Ping Lan, Lei Wang, Xiang Gao, Teng‐Hui Ma

**Affiliations:** ^1^ Department of Radiation Oncology The Sixth Affiliated Hospital Sun Yat-sen University Guangzhou People’s Republic of China; ^2^ Department of Colorectal Surgery The Sixth Affiliated Hospital Sun Yat‐Sen University Guangzhou People’s Republic of China; ^3^ Guangdong Institute of Gastroenterology Guangzhou People’s Republic of China; ^4^ Guangdong Provincial Key Laboratory of Colorectal and Pelvic Floor Diseases The Sixth Affiliated Hospital Sun Yat‐Sen University Guangzhou People’s Republic of China; ^5^ Department of Gastroenterology Guangdong Provincial Key Laboratory of Colorectal and Pelvic Floor Diseases The Sixth Affiliated Hospital Sun Yat‐Sen University Guangzhou People’s Republic of China

**Keywords:** FOWARC clinical trial, interval between RCT and surgery, pathologic complete regression, radiation proctitis, rectal cancer

## Abstract

**Objective:**

The aim of this study was to evaluate the effect of the interval between CRT and surgery on radiation proctitis, the pathologic response, and postoperative morbidity.

**Methods:**

This was a cohort study from a phase III, randomized controlled trial (FOWARC study, NCT01211210). Data were retrieved from the leading center of the trial. Patients were divided into the short‐interval (≤7 weeks) group and the long‐interval (>7 weeks) group. The rate of radiation proctitis, pathologic complete regression (pCR) and morbidities were calculated for each group. Multivariate analysis was used to verify the impact of interval on radiation proctitis.

**Results:**

Surgery was performed in 60 patients after an interval of ≤7 weeks and in 97 patients after an interval of >7 weeks. The two groups according to interval were comparable in terms of baseline demographic and clinicotherapeutic characteristics. Radiation proctitis was identified by imaging in 9 (15.0%) patients in short‐interval group and in 31 (32.0%) patients in long‐interval group (*P* = .018). Multivariate analysis confirmed the correlation between long interval and radiation proctitis (*P* = .018). The long interval was significantly associated with longer median operation time compared to the short interval (*P* = .022). The rates of pCR and postoperative complications were not different between two groups.

**Conclusions:**

A longer interval after CRT may be associated with higher rate of radiation proctitis and longer operation time. Moreover it did not increase the rate of pCR.

## Graphical abstract

A longer interval after CRT may be associated with higher rate of radiation proctitis and more difficult surgical resection. Moreover it did not increase the rate of pCR.

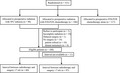

## 1 INTRODUCTION

Preoperative chemoradiotherapy (CRT) is associated with better local control and higher rates of sphincter preservation compared to postoperative CRT.^1^, ^2^, ^3^ Thus, neoadjuvant CRT followed by total mesorectal excision (TME) has become the standard of care for patients with locally advanced rectal cancer.^4^

Radiation‐induced bowel toxicity is quite common during pelvic CRT,^5^, ^6^, ^7^ which is a major problem because of its negative impact on treatment compliance, quality of life of patients and associated additional economic burden to the already costly process of cancer care.^8^ In addition, acute radiation‐proctitis was reported to predict late symptomatic proctitis.^9^ Radiation proctitis is rarely investigated in rectal cancer since both diseases manifest similar symptoms. Even though it is potentially underestimated in clinical practice and there is lack of comprehensive criteria for the diagnosis of radiation‐induced bowel injury, it was reported that radiation proctitis was a normal adverse event for rectal cancer patients receiving pelvic radiation.^10^ Considering its potential impact on quality of life of patients and surgery complications such as anastomotic leakage,^11^, ^12^ it is crucial to identify possible risk factors of radiation proctopathy.

The rationale for time‐scale effect of radiation is based on the phenomenon‐DNA damage occurs during irradiation, but cellular lysis occurs within the next weeks.^13^, ^14^, ^15^ Therefore, the interval between CRT and surgery not only affects tumor regression, but also influences the probability of normal tissue complications. In rectal cancer, a delay before surgery of 6‐8weeks after radiotherapy is standard.^16^ A recently published randomized trial from the French GRECCAR group showed that waiting for 7 weeks after chemoradiotherapy achieved lower surgical morbidity without compromising the rate of pCR than for 11 weeks.^17^ However, no study to date has investigated whether optimal interval have an impact on radiation proctitis.

To evaluate the effect of the interval between neoadjuvant CRT and surgery on radiation proctitis and clinical outcome, we conducted a post hoc analysis of the data from the leading center in a phase III randomized clinical trial (FOWARC study: NCT01211210).^18^

## 2 PATIENTS AND METHODS

### 2.1 Patients

FOWARC is a multicenter, open‐label, randomized, phase III trial which was registered on the clinicaltrials.gov Web site with the identifying number NCT01211210.^18^ Eligibility criteria was illustrated in our previous study in detail. The trial was approved by ethics committees of all of the participating centers. From June 2010 to February 2015, 321 patients from the leading center were randomly assigned to receive neoadjuvant radiation and 5‐FU infusion (arm A), neoadjuvant radiation and FOLFOX chemotherapy (arm B), or neoadjuvant FOLFOX chemotherapy alone (arm C). Two hundred and six patients with radiotherapy from arm A and arm B were included in our study since the primary objective of the present study was radiation proctitis. An interval of 6‐8 weeks is generally accepted and has become routine practice. The median interval was 7 weeks, which was used as a cutoff value in our study. Therefore, patients were divided into two groups according to the interval between CRT and surgery of 7 weeks.

The following data were reviewed from our prospectively entered database: gender, age, body mass index (BMI), comorbidity, American Society of Anesthesiologists (ASA) classification, clinical T and N stage, pretreatment distance from the anal verge, the interval between CRT and surgery, intraoperative complications, operative time, estimated blood loss, length of hospital stay, postoperative morbidity and mortality, and final pathologic stage.

### 2.2 Treatment

Long‐course fractionated radiation was delivered at 1.8 to 2.0 Gy daily fractions administered five times weekly for a total of 23 to 28 fractions over 5 to 6 weeks. The clinical target volume included the mesorectum and pelvic lymphatic area. Patients in arm A received preoperative treatment with five cycles of infusional fluorouracil (leucovorin 400 mg/m^2^ intravenously followed by fluorouracil 400 mg/m^2^ intravenously and fluorouracil 2.4 g/m^2^ by 48‐hours continuous intravenous infusion) every 2 weeks. Patients in arm B received the same treatment as the fluorouracil‐radiotherapy group plus oxaliplatin 85 mg/m^2^ intravenously on day 1 of each chemotherapy cycle every 2 weeks. Adjuvant chemotherapy with the same regimen as the neoadjuvant chemotherapy started within 6 weeks after surgery.

### 2.3 Definition of outcomes

The primary endpoint of this study was radiation proctitis. Radiation enteropathy is generally classified as acute when it occurs within 3 months of radiation therapy, or chronic when it occurs more than 3 months after radiation therapy. Therefore, all radiation proctitis in our study was acute proctitis. Radiation proctitis in rectal cancer was defined as rectal injury after pelvic radiation without considering patients’ reported discomfort since both diseases manifest similar symptoms. MRI or CT was performed within 3 days before surgery and was necessary for the diagnosis of radiation proctitis (Figure S1), which manifested as circumferential thickening of the rectal wall with mural stratification, diffuse edema of mesorectum and pelvic soft tissue, and accompanying alterations of the sigmoid colon within the radiation field. Colonoscopy was partly used as a supplement revealing diffuse mucosal edema, erythema, paleness, friability, and bowel stiffness within the pelvis. Two experienced radiologists independently evaluated all the scans of pelvic MRI and CT. All disagreements were resolved by consensus and by the assessment of complementary colonoscopy. Radiologists and endoscopist were blinded to possible symptoms and the interval.

The secondary outcomes included pathologic tumor regression and postoperative complication. Postoperative specimens were examined by two pathologists specialized in colorectal cancer. Pathologic complete remission (pCR) (ypT0N0) was defined as absence of viable carcinoma cells in the operative specimen, including primary tumor and lymph nodes. The occurrence of surgical complications within 90 postoperative days was defined as postoperative complications.

### 2.4 Statistical analysis

Patients completed radiotherapy were included in the post hoc analysis. The *χ* ^2^ test and Fisher exact test was used to compare patients with an interval of ≤7 and >7 weeks. The unpaired *t* tests or the Mann‐Whitney U test was used for continuous variables. A two‐sided *P* ≤ .05 was considered statistically significant. To verify the effect of interval on radiation procotitis, multiple logistic regression models also included chemotherapy, pelvic node irradiation and coexisting comorbidities such as diabetes, vascular disease and inflammatory bowel disease (IBD). Data were analyzed by Statistical Package for the Social Science (SPSS) 19.0 for Windows (SPSS, Inc, Chicago, IL).

## 3 RESULTS

### 3.1 Patients and radiation proctitis

**Figure 1:**
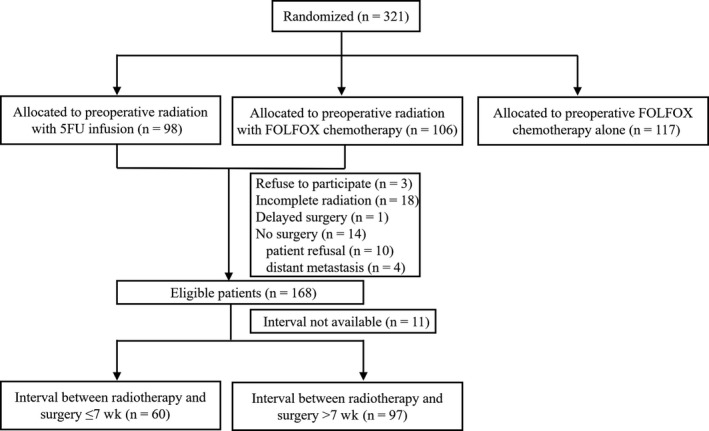
Flow diagram of the study. 5‐FU = 5‐fluorouracil; mFOLFOX6 = modified regimen with fluorouracil, leucovorin, and oxaliplatin

Of the 204 patients randomly assigned to the arm A and arm B, 3 patients were not included because they withdrew consent to participate. The baseline characteristics of remaining 201 patients were shown in supplementary Table 1. Eighteen of 201 patients have not completed radiotherapy and fourteen of them have not received surgery because of refusal or progressed disease. In addition, 1 patient postponed surgery for almost one year and the interval between CRT and surgery cannot be obtained in 11 patients. Therefore, a total of 157 patients were eligible to be analyzed (Figure 1). Surgery was performed in 60 patients after an interval of ≤7 weeks (median 44 days, range 26‐49 days) and in 97 patients after an interval of >7 weeks (median 55 days, range 50‐78 days). The baseline demographic and clinicotherapeutic characteristics were comparable in these two groups (Table 1).

**Table 1:** Demographic and clinicotherapeutic characteristics of 157 patients

Characteristic	Interval ≤ 7 wks (n = 60)	Interval > 7 wks (n = 97)	*P* value
Interval, days	44 (26‐49)	55 (50‐78)	<.001^a^, ^b^^,^ a, b
Radiation proctitis	9 (15.0)	31 (32.0)	0.018^a^, ^b^^,^ c
Gender			0.183^c^
Women	17 (28.3)	31 (32.0)	
Men	43 (71.7)	66 (68.0)	
Age, y	55 ± 10	52 ± 12	0.068^d^
BMI, kg/m^2^	22.7 ± 3.1	22.1 ± 2.6	0.201^d^
ASA score			0.910^b^
1	7 (11.7)	13 (13.4)	
2	52 (86.7)	83 (85.6)	
3	1 (1.6)	1 (1.0)	
Clinical T classification			0.240^b^
cT2	2 (3.3)	4 (4.1)	
cT3	47 (78.3)	64 (66.0)	
cT4	11 (18.4)	29 (29.9)	
Clinical N classification			0.462^c^
cN0	15 (25.0)	21 (21.6)	
cN1	20 (33.3)	42 (43.3)	
cN2	25 (41.7)	34 (35.1)	
Distance of tumor from anal verge, cm			0.663^b^
>10	3 (5.0)	8 (8.2)	
5‐10	31 (51.7)	44 (45.4)	
<5	26 (43.3)	45 (46.4)	
Chemotherapy regimen			0.624^c^
5‐FU	30 (50.0)	41 (42.3)	
FOLFOX	30 (50.0)	56 (57.7)	

Note

Data are median (range), n (%) or mean ± SD.

Abbreviations: BMI, body mass index; ASA, American society of Anesthesiologists; 5‐FU, 5‐fluorouracil; FOLFOX, 5‐Fluorouracil + oxaliplatin + leucovorin.

a Statistically significant.

b Data were calculated using the Mann‐Whitney U test.

c Data were calculated using the χ^2^ test.

d Data were calculated using the *t* test.

Data were calculated using the Fisher exact test.

### 3.2 Surgical characteristics and postoperative course

Surgical characteristics were detailed in Table 2. Fifty‐two (86.7%) patients underwent sphincter‐preservation operation in the short‐interval group and 86 (88.7%) patients in the long‐interval group (*P* = .71). The long interval was significantly associated with longer median operation time compared to the short interval (250 vs 232 minutes, *P* = .022). The estimated blood loss was not significantly influenced by the interval between radiotherapy and surgery.

**Table 2:** Surgical characteristics and postoperative course

Characteristic	Interval ≤ 7 wks (n = 60)	Interval > 7 wks (n = 97)	*P* value
Type of surgical procedure			.71^b^
Low anterior resection	52 (86.7)	86 (88.7)	
Abdominoperineal excision (APE)	8 (13.3)	11 (11.3)	
Defunctioning ileostomy	46 (76.7)	76 (78.4)	.987^b^
Median operation time, minutes	232 (120‐445)	250 (105‐435)	.022^a^, ^c^^, ^ a, c
Estimated blood loss, ml	100 (10‐350)	100 (20‐2000)	.494^a^, ^c^
Mean units of packet blood			
Postoperative complications	15 (25.0)	33 (34.0)	.233
Anastomotic leakage	8 of 52 (15.4)	13 of 86 (13.4)	.966^b^
Grade B	5 (9.6)	9 (10.5)	
Grade C	3 (5.8)	4 (2.9)	
Perineal complications after APE	2 of 8 (12.5)	6 of 11 (54.5)	.352^d^
Urinary complications	3 (5.0)	7 (7.2)	.743^d^
Uroschesis	2 (3.3)	6 (6.2)	.711^d^
Infections	1 (1.7)	6 (6.2)	.252^d^
Postoperative ileus	4 (6.7)	6 (6.2)	1.00^d^

Note

Data are median (range) or n (%).

a Statistically significant.

b Data were calculated using the χ^2^ test.

c Data were calculated using the Mann‐Whitney U test.

d Data were calculated using the Fisher exact test.

### 3.3 Pathologic response

pCR and other pathologic characteristics are shown in Table 3. Forty‐one (26.1%) patients achieved pCR. The rate of pCR was not affected by the length of interval. In addition, downstaging for both tumor and node category did not differ significantly between two groups.

**Table 3:** Pathologic characteristics

Characteristic	Interval ≤ 7 wks (n = 60)	Interval > 7 wks (n = 97)	*P* value
ypT stage			.949^a^
T0	16 (26.7)	29 (29.9)	
T1	3 (5.0)	3 (3.1)	
T2	15 (25.0)	21 (21.6)	
T3	22 (36.7)	37 (38.1)	
T4	4 (6.6)	7 (7.2)	
ypN stage			.340^a^
N0	49 (81.7)	84 (86.6)	
N1	5 (8.3)	9 (9.3)	
N2	6 (10.0)	4 (4.1)	
pCR (ypT0N0)	15 (25.0)	26 (26.8)	.803^b^
T downstaging (ypT < cT)	38 (63.3)	65 (67.0)	.637^b^
N downstaging (ypN < cN)	38 (63.3)	67 (69.1)	.458^b^
Tumor regression grade			.561^a^
0	15 (25.0)	29 (29.9)	
1	20 (33.3)	35 (36.1)	
2	24 (40.0)	29 (29.9)	
3	1 (1.7)	4 (4.1)	
Tumor size, cm	4.3 (0‐9.0)	4.0 (0‐8.7)	.425^c^
Number of harvested lymph nodes	9 (0‐24)	8 (0‐25)	.589^c^
Number of positive lymph nodes	0 (0‐15)	0 (0‐17)	.746^c^
CRM involved	2 (3.3)	2 (2.1)	.495^a^
Lymphovascular invasion	5 (8.3)	2 (2.1)	.108^a^
Perineural invasion	1 (1.7)	4 (4.1)	.650^a^

Note

Data are median (range) or n (%).

pCR, pathologic complete remission; CRM, circumferential resection margin.

a Data were calculated using the Fisher exact test.

b Data were calculated using the χ^2^ test.

c Data were calculated using the Mann‐Whitney U test

### 3.4 Radiation proctitis

Radiation proctitis was identified by imaging in 9 (15.0%) patients in short‐interval group and in 31 (32.0%) patients in long‐interval group (*P* = .018). Multivariate analysis showed that patients in long‐interval group were associated with higher rates of radiation proctitis (*P* = .018). Seventeen patients (10.8%) were treated within an interval of <6 weeks and 41 patients (26.1%) after an interval >8 weeks. The incidence of radiation proctitis was significantly higher in long‐interval group than short‐interval group (*P* = .018). Table 4 shows the univariate analysis of variables with clinical implications. Logistic regression models included chemotherapy, pelvic node irradiation and coexisting comorbidities such as diabetes, vascular disease, and inflammatory bowel disease (IBD). And we identified the interval as an independent risk factor for radiation proctitis (*P* = .021) with HR:2.663 (95%CI: 1.157‐6.129) (S Table 2).

**Table 4:** Univariate analysis of factors associated with radiation proctitis in patients receiving chemoradiotherapy

Variable	No. of radiation proctitis/total patients (%)	*P* value
Interval		.018^a^^,^ b
≤7 wks	9/60 (15.0)	
>7 wks	31/97 (32.0)	
Comorbidities		.619^b^
Yes	6/20 (30.0)	
No	34/137 (24.8)	
Smoking		.165^b^
Yes	7/40 (17.5)	
No	11/117 (9.4)	
Pelvic node irradiation		.716^c^
Yes	3/10 (30.0)	
No	37/147 (25.2)	
Chemotherapy regimen		.689^b^
5‐FU	17/71 (23.9)	
FOLFOX	23/86 (26.7)	
Gender		.494^b^
Women	11/50 (22.0)	
Men	29/107 (27.1)	
Age		.284^b^
≤60	24/105 (22.9)	
>60	16/52 (30.8)	
AJCC stage		
II	7/35 (20.0)	.399^b^
III	33/122 (27.0)	

Note

5‐FU, 5‐fluorouracil; FOLFOX, 5‐fluorouracil + leucovorin + oxaliplatin; AJCC, American Joint Committee on Cancer (7th edition).

a Statistically significant.

b Data were calculated using the χ^2^ test.

c Data were calculated using the Fisher exact test.

## 4 DISCUSSION

This is a post hoc study in a consecutive group of patients with locally advanced rectal cancer treated with long‐course neoadjuvant CRT from a prospective phase III clinical trial. This study aimed to investigate the impact of different intervals between neoadjuvant CRT and curative surgery for locally advanced rectal cancer on radiation proctitis, pathologic response, and postoperative morbidity.

Patients were divided into two groups according to the neoadjuvant CRT‐surgery interval: short‐interval group (≤7 weeks) and long‐interval group ( >7 interval). In accordance with our study, some prospective clinical trial and retrospective studies also applied an interval of 7 weeks as cutoff value.^17^, ^19^, ^20^ The present study showed that a longer interval was associated with more operative time and higher rates of radiation proctitis. However, we found that a longer interval was not related with pCR rate, postoperative morbidities. The result of pCR are inconsistent with those of previously published retrospective studies.^19^, ^20^, ^21^ This discrepancy may be explained by our high pCR rate. Our 26.1% pCR rate was higher than the rate reported in previous publications because of the introduction of FOLFOX regimen and IMRT^18^ and patients received a full dose of mFOLFOX6 regimen and had a high rate of treatment compliance to receive full‐dose radiation. Furthermore, the addition of chemotherapy during the waiting period may improve the pCR and tumor downstaging rates. Since a “glass ceiling” regard to pT category exists for pCR rate, waiting longer cannot change the intrinsic characteristics of the tumor. Similarly, the result from a recent multicenter, randomized, phase III trial (GRECCAR‐6) was consistent with our result which showed that a longer interval did not increase the rate of pCR.^17^

Previous studies have showed that tumor regression and radiation‐induced injury are a time‐dependent phenomenon.^22^, ^23^, ^24^ Thus, we found that a longer interval was significantly associated with higher rate of radiation proctopathy, though it was not related with the rates of downstaging and pCR due to the intensive treatment strategy between two groups. To the best of our knowledge, the present study was the first to report that a longer interval between CRT and surgery is associated with a higher rate of radiation proctopathy. The possible reason is that most of previous studies do not specifically report on rates of radiation proctitis because its definition is easy to be blurred, and its symptoms is easy to be obscured with rectal cancer. Therefore, radiation proctitis in rectal cancer was cautiously defined by imaging complementary endoscopy in the present study due to the lack of uniform criterion or consensus and obscure symptoms.^25^ Our present study showed that the long interval was significantly associated with longer median operation time compared to the short interval (*P* = .022) and the long interval subgroup had higher rates of postoperative complications (25% vs. 34%) than the short interval subgroup. Furthermore, the rate of severe low anterior syndrome (LARS) was higher in long interval group than short interval group (85% vs 75%). These findings may be associated with the higher rates of acute radiographic radiation proctitis in the long interval group. Due to the relative limited samples in our study, differences of postoperative complications rates and severe LARS rate did not achieve statistical significance. However, it gives us a hint to urge caution of radiation proctitis and postoperative complication in the long interval group. But we would not advocate altering treatment decision on the basis of our findings, which need to be confirmed by other analyses of larger sample size or by prospective, randomized, controlled clinical trials. Smoking was reported to be a crucial factor to be related with higher rates of radiation toxicity especially enteritis.^26^, ^27^ Although the present study showed higher incidence of radiation proctitis in patients who are smoking, the difference was not significant because of relatively small sample size.

Although the present study was a post hoc study from a phase III randomized clinical trial and patients received standard treatment, it may have potential bias due to its retrospective nature. However, the demographics and clinical characteristics were comparable between the two groups. Furthermore, this post hoc study from a clinical trial only indicated the association between radiation proctitis and interval of chemoradiotherapy and surgery. Therefore, further well designed prospective study with larger sample size was in great need to verify our result and identify their certain correlation.

In conclusion, the present study showed that a neoadjuvant‐surgery interval >7 weeks was correlated with higher rate of radiation proctitis and longer operative time and do not increase the rate of pCR. Waiting more than 7 weeks after neoadjuvant radiochemotherapy, surgeons should pose caution in radiation proctitis.

## CONFLICT OF INTEREST

We declare that we have no conflicts of interest.
