# Supplementary figures and images for: Effect of interval between preoperative radiotherapy and surgery on clinical outcome and radiation proctitis in rectal cancer from FOWARC trial
Source: Cancer Med. 2019 Dec 12;9(3):912–9. doi: 10.1002/cam4.2755 (PMC6997091; doi:10.1002/cam4.2755)

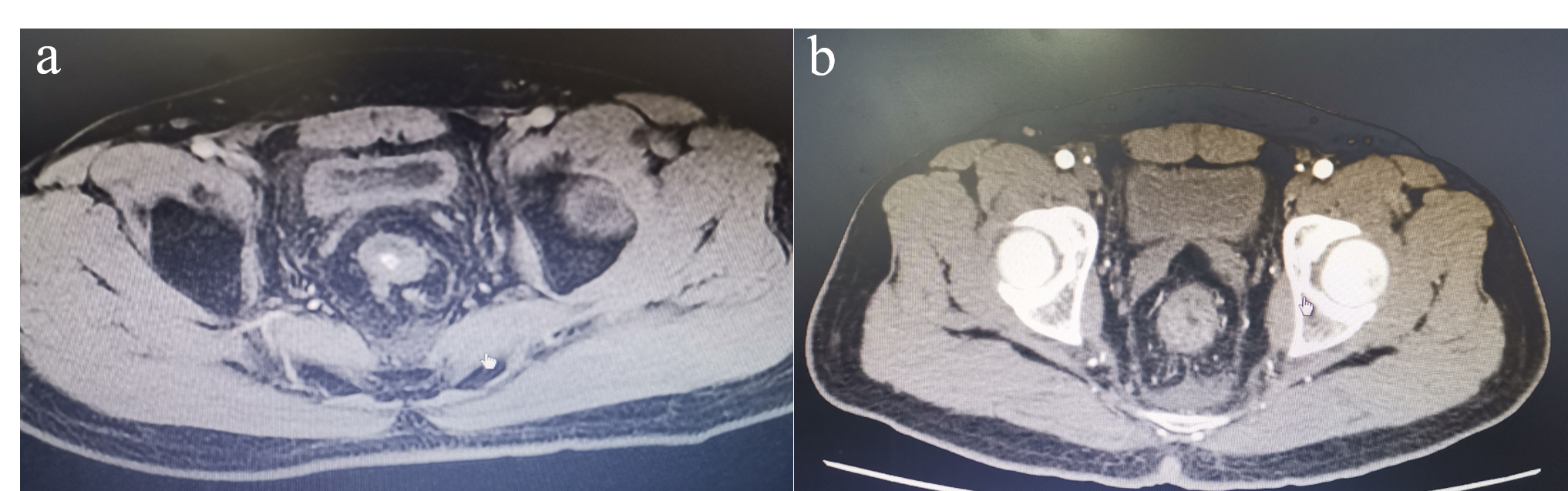

Supplement: Supplementary file 1 [file CAM4-9-912-s001.tif]
