# Supplementary material for: Effect of interval between preoperative radiotherapy and surgery on clinical outcome and radiation proctitis in rectal cancer from FOWARC trial
Source: Cancer Med. 2019 Dec 12;9(3):912–9. doi: 10.1002/cam4.2755 (PMC6997091; doi:10.1002/cam4.2755)

| **Characteristic** | **Neoadjuvant RT+5-FU (n=97)** | **Neoadjuvant RT +FOLOX (n=104)** |
| --- | --- | --- |
| Gender |  |  |
| Women | 34 (35.1) | 30 (28.8) |
| Men | 63 (64.9) | 74 (71.2) |
| Age, y | 55.8±12.2 | 51.2±11.3 |
| BMI, kg/m^2^ | 22.3±3.2 | 22.4±2.7 |
| AJCC stage |  |  |
| II | 23 (23.7) | 20 (19.2) |
| III | 74 (76.3) | 84 (80.8) |
| Tumor size, cm | 4.4±1.7 | 4.4±1.4 |
| Distance of tumor from anal verge, cm |  |  |
| >10 | 2 (2.1) | 4 (3.8) |
| 5-10 | 55 (56.7) | 60 (57.7) |
| <5 | 40 (41.2) | 40 (38.5) |

**Table 1. Baseline characteristics of 201 patients from arm A and arm B**

Data are n (%) or mean ± SD.

BMI= body mass index; AJCC = American Joint Committee on Cancer (7th edition); preop = preoperative; 5-FU = 5-ﬂuorouracil; FOLFOX = ﬂuorouracil, leucovorin, and oxaliplatin; RT = radiotherapy

**S Figure 1.** MRI (a) and CT (b) images for radiation proctitis.


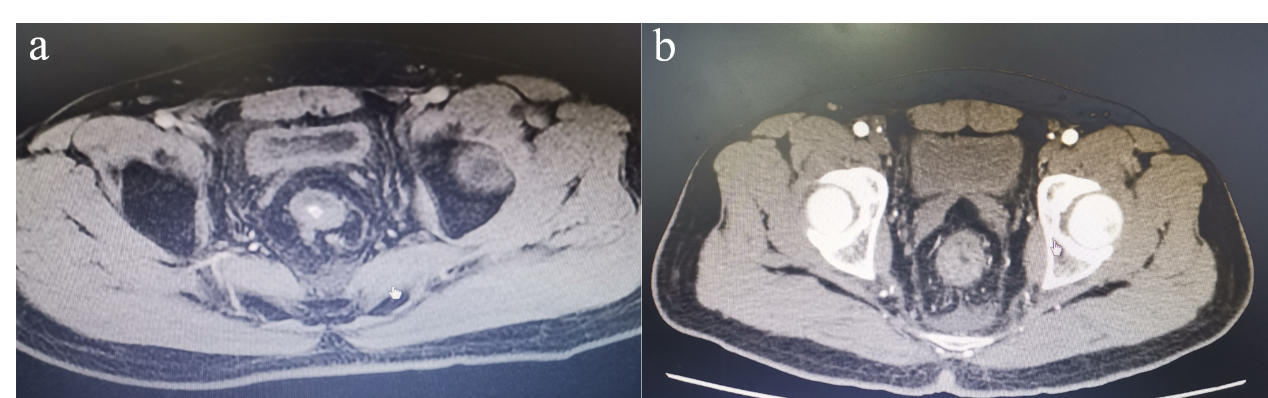

Supplement: Supplementary file 2 [file CAM4-9-912-s002.docx]
